# The Influence of Exercise on Growth

**Published:** 1897-03

**Authors:** 


### The Influence of Exercise on Growth.

The usual effects of properly-applied exercise not only produce a sense of exhilaration and a feeling of well-being, but when conducted along certain lines and governed by scientific principles it brings about certain remote and permanent effects. In the Bible it is asked who can, by taking thought, add one cubit to his stature.

Surgeon Henry G. Beyer, of the United States Navy, has given the subject considerable thought, and he thinks he has added markedly to the stature of many cadets who took exercise according to his directions. His careful and practical article in the *Journal of Experimental Medicine* shows how systematic exercise adds not only to the height, but to the weight and strength.

He first studied the normal growth tables from the sixth to the twenty-second year. These tables were obtained from various and reliable sources. He then began the task of measuring each cadet passing under his care, and he recorded the comparative measurements on entrance, during a carefully-graded course of exercise, and at the end of this period. His results are not only valuable, but the manner in which he so intelligently uses his statistics is extremely praiseworthy. He found the most rapid growth between the fifteenth and twenty-first year to be in the fifteenth and next in the sixteenth year. Between sixteen and twenty years he found that properly-administered gymnastic exercise showed an increase in height above the normal growth of 26.6 millimeters, or a little more than one inch. If the height is increased, it follows, naturally, that in the large majority the weight and the strength are also increased.

Height is the most important consideration in this kind of investigation, because any agent influencing growth in height in man influences growth in bone. Growth practically ceases at twenty-one, while increase in weight and strength may be obtained almost indefinitely. The increase in strength from this kind of exercise was almost marvelous, and he is inclined to think that almost any healthy young man can become a "phenomenal giant" if he exercises systematically and regularly.

The kind of exercise needed is not that which will alone increase the weight and strength, but also that which will increase the height, and then the other form of increase will follow as a matter of course.

This work of Dr. Beyer is especially interesting as showing why the young child should not be allowed to "just grow up," like Topsy, but should be given a systematic and regular course of training as laid down by this writer, so that a race of strong and tall men may have the opportunity to marry and bring into the world strong and healthy offspring to counteract the many degenerating and enervating effects of the style of living of the present day.—*Maryland Med. Journal.*

